# Users’ perspectives on a demonstration to increase shared access to older adults’ patient portals

**DOI:** 10.1186/s12913-025-12755-0

**Published:** 2025-04-23

**Authors:** Vadim Dukhanin, Jennifer L. Wolff, Kelly Gleason, Deborah Wachenheim, Liz Salmi, Matthew J. Gonzales, Marianne Parshley, Sara Epstein, Supriya Mohile, Timothy W. Farrell, Mark A. Supiano, Catherine M. DesRoches, the Shared Access Project Team, Danielle Peereboom, David L. Roth, Mingche M. J. Wu, Doug Niehus, Caroline Reay, Jill DelVecchio, Kathleen Fear, Martha Kenyon, Saloni Sharma, Diane Tadehara, Shawn Wells

**Affiliations:** 1 Department of Health Policy and Management, Johns Hopkins Bloomberg School of Public Health, Baltimore, MD USA; 2 Johns Hopkins University School of Nursing, Baltimore, MD USA; 3 OpenNotes, Division of General Medicine, Department of Medicine, Beth Israel Deaconess Medical Center, Boston, MA USA; 4 The Institute for Human Caring at Providence, Gardena, CA USA; 5 Providence Medical Group, Portland, OR USA; 6 Wilmot Cancer Institute, University of Rochester Medical Center, Rochester, NY USA; 7 Division of Geriatrics, Department of Internal Medicine, Spencer Fox Eccles School of Medicine, University of Utah, Salt Lake City, UT USA; 8 Salt Lake City Geriatrics Research, Education, and Clinical Center, Veterans Administration, Salt Lake City, UT USA; 9 Department of Medicine, Harvard Medical School, Boston, MA USA

**Keywords:** Patient portal, Electronic health record, Care partners, Stakeholder engagement, Patient engagement, Family engagement, Older adults, Digital platform, Awareness

## Abstract

**Background:**

Many patient portals allow patients to authorize a care partner to use the portal on their behalf, with evidence suggesting a range of benefits to patients, care partners, and clinicians. Shared or proxy access aligns with patient- and family-centered care and supports care partners’ legitimacy and identification by clinicians in patient portal interactions. As shared access uptake remains low, the Coalition for Care Partners (https://coalitionforcarepartners.org) and three healthcare delivery organizations co-designed an initiative promoting shared access to the patient portals of older adults.

**Objective:**

To evaluate an initiative’s demonstration through users’ perspectives.

**Design:**

The 12-month demonstration was launched at five clinics (geriatric oncology, primary care, and geriatric medicine) across the three organizations. Clinicians and staff were interviewed mid- and post-demonstration via focus groups and individually; clinic patients and care partners responded to an anonymous post-demonstration online portal survey.

**Participants:**

Demonstration users included established patients from the five clinics and their care partners, as well as clinic physicians, nurses, social workers, care managers, patient-facing staff, administrators, and information technology specialists.

**Approach:**

We followed the Consolidated Framework for Implementation Research to develop interview guides and inform our analysis of the survey’s open-ended responses and interview transcripts. We analyzed 11 focus groups, 10 individual interviews, and 392 patients’ and 79 care partners’ survey responses employing rapid assessment procedures methodology.

**Key results:**

The demonstration was appropriate, useful, routinized in the clinics, and well received by patients and care partners. The demonstration was perceived as undemanding and low-cost, utilizing existing infrastructure and organizational processes. Facilitators included alignments of shared access with clinics’ practice and philosophy, organizations’ policies, and needs of patients and care partners. Identified barriers included clinicians’ competing priorities and patients’ and care partners’ low awareness and motivation for using shared access.

**Conclusions:**

The Coalition for Care Partners can spread this appropriate, useful, undemanding, and low-cost initiative. Further efforts might be supported by policies that ensure that shared access benefits are bolstered, potential harms of unidentifiable access are emphasized, and registration is conceptualized as an opt-out versus opt-in process.

**Supplementary Information:**

The online version contains supplementary material available at 10.1186/s12913-025-12755-0.

## Introduction

Patient portals are widely used in healthcare delivery and provide patients with access to their medical records, including clinical notes, visit summaries, test results, and tools to schedule appointments, refill medications, and communicate with their care teams [1]. Many patient portals have a functionality allowing patients to “share access” to their patient portal account (also known as “proxy access”) by authorizing a person(s) via a registration process providing these persons with individual login information and making them identifiable as delegates when they access the patient portal [2, 3]. Thus, shared access identifies and confirms that these care partners - family members, friends, patient advocates, or others - are authorized by the patient to use the portal on their behalf. Previous studies established that shared access is desired by patients [4–7], aligns with patient- and family-centered care [8, 9], and is welcomed by clinicians who are able to identify care partners when interacting with them on the patent portal [10]. By clarifying and fulfilling patients’ desires to involve one or more care partners, shared access supports greater legitimacy of, and convenience for, care partners in their healthcare interactions, and facilitates stronger trilateral partnerships between patients, care partners, and healthcare professionals [11, 12]. Shared access facilitates more explicit and purposeful engagement of care partners which benefits quality and safety of care, increases patient and care partner satisfaction, and boosts care partner confidence in their ability to comanage care [10, 11]. 

Despite identified benefits, awareness and use of shared access are low [13–16], and the registration process is often not straightforward [6, 17, 18]. Recent studies reveal that care partners commonly resort to accessing patient portals using patient credentials rather than securing their own [14–16, 18–20]. This obscures clinicians’ understanding of whether and when care partners are involved in electronic interactions, raises privacy and confidentiality concerns, and leaves care delivery organizations without the ability to discern when a care partner (rather than the patient) is messaging clinicians, responding to patient assessments, or uploading legal documents, such as advance directives [10]. To address this problem, a partnership between three healthcare organizations and the Coalition for Care Partners [21] launched a first-of-its-kind initiative with the goal of overcoming barriers to shared access awareness and registration [22]. The Coalition for Care Partners (https://coalitionforcarepartners.org) has a mission to help develop and spread health information technologies that support care partners and alleviate stress as they work to deliver care, and all three participating healthcare organizations joined the Coalition to work on this initiative. The demonstration of this initiative took place July 2022-October 2023 in five clinics operated by the three organizations. Mixed results in terms of care partners’ registration and use of the patient portals during the demonstration, and the current paucity of similar studies, necessitated its further multifocal assessment [23]. Understanding appropriateness and usefulness, practicality and costs, and barriers and facilitators of this initiative will also inform future routinization of shared access in mainstream healthcare practice. Thus, we aimed to evaluate the demonstration to increase shared access to older adults’ patient portals by gathering perspectives of organizational and clinic personnel and patients and care partners served in the participating clinics.

## Methods

### Overview

The initiative was co-designed with the 3 organizational partners via numerous engagement activities with patients and care partners, clinicians and clinic staff, medical informatics teams, marketing and communications staff, administrators, and funders and thought leaders, and is previously described in detail [22]. Co-design is a participatory, intentionally inclusive and collaborative activity that mandates that researchers and organizations focus on understanding “end users” and engaging them directly in problem-solving and the development of solutions. The 12-month demonstration of the initiative included the deployment of co-designed brochures and posters, organization-specific web pages detailing shared access registration processes, clinician and staff talking points about shared access, and staff tip sheets that outline shared access registration steps. At one clinic, new patients received the brochure as a part of a new patient packet mailed to their homes. At three clinics, clinic staff provided information about shared access during appointment reminder calls to older patients if care partner involvement in the upcoming visit was mentioned. At all five clinics, the brochures were offered at the check-in desks by the staff, and at two clinics, front-desk staff reminded patients and care partners about the brochure at checkout. At three clinics, medical assistants raised the topic of shared access and offered brochures during rooming or in telehealth visit communications. At all clinics, clinicians were encouraged to raise and endorse the benefits of shared access, offering older patients a brochure or asking staff to provide it. During in-person or telehealth visits, clinicians utilized a smart phrase that a one-paragraph description about shared access in the patient’s after-visit summary and visit note. This smart phrase was used to generate the same description for the patient portal messaging. At telehealth visits, clinicians shared the electronic version of the brochure. At three clinics, patient service specialists were empowered to initiate conversations about shared access during telephone calls. Finally, at a patient’s request, a medical assistant, registered nurse, or social worker could help the patient register their care partners for shared access while they were at the clinic after the visit. The educational materials were accompanied by implementation toolkits specifying and reinforcing workflows involving both in-person and telehealth visits [22]. During the co-designing of educational materials it was decided that the target audience for the materials was older adult patients, rather than care partners, reflecting the need to center the autonomy, dignity, and privacy of older adults. Each clinic had a staff or clinician champion, or both who supported the demonstration. The demonstration was also supported by information technology specialists who were responding to requests from staff, patients, and care partners to help with shared access registration as part of their routine workflow.

The demonstration took place at the following 5 clinics: a geriatric oncology clinic (organization 1, clinic A); 3 primary clinics located in the same city (organization 2, clinics B, C, and D); and a geriatric medicine clinic (organization 3, clinic E). See Table 1 for characteristics of these organizations and clinics. The organizations were selected through a rigorous process identifying those that were early adopters of sharing medical visit notes with patients and had stable patient portal platforms, engaged and innovative chief medical information officers, active Patient and Family Advisory Councils (PFACs), and capabilities for reporting their shared access use. Clinics offered web-based (initiated by patients via the patient portal) and in-person options of shared access registration. At all organizations, the clinical information on the patient portal was only in English.

**Table 1:** Characteristics of organizations participating in the demonstration and focus groups and interviews with those organizations’ clinic personnel

	Organization 1	Organization 2	Organization 3
Total number of physicians in the organization	2,300+	14,000+	1,600+
Total number of organization’s hospitals	6	55+	2
Participating clinics	1 (clinic A), geriatric oncology	3 (clinics B, C, and D), in 1 city, primary care	1(clinic E), geriatrics
Number of clinicians involved	8	34, in total	12
Number of staff involved	5	93, in total	15
Clinic, geographic location	Urban	Urban	Urban
Clinic, total visits of patients age 65 years and older, annually	500+	30,000+	7,500+
Mid-demonstration focus groups	None	3, at clinics’ regular all staff and clinician meetings	1, with staff; and 1, with clinicians, at clinic’s regular meetings
Post-demonstration focus groups	1, at clinic’s regular all staff and clinician meeting	3, at clinics’ regular all staff and clinician meetings	1, with staff; 1, with clinicians at clinic’s regular meetings; and 1, with convened care managers and social workers
Post-demonstration individual interviews	1, with a physician clinic leader; 1, with a physician assistant clinic’s champion; 1, with a health information technology specialist; and 1, with a social worker	1, with a physician clinic’s champion; and 1, with a health information technology specialist	1, with a physician; 1, with a nurse clinic’s champion; and 1, with a health information technology specialist

The evaluation strategy was also co-designed, iteratively identifying and clarifying outcomes that would be feasible to collect and could help with understanding the value of shared access. Our evaluation strategy included both qualitative and quantitative measures of implementation. Measures of care partners’ registration and use of the patient portals and measures of patients’ and care partners’ awareness of shared access and preferred use are described elsewhere [23]. Overall, shared access account activation at the beginning of the demonstration was higher than reported in the literature (< 3% of patients authorized at least one care partner) [16] and varied across clinics (5.0% for primary care, 24.6% for geriatric medicine, and 19.7% for geriatric oncology). This variation was expected given the varying degree of care partners’ involvement in primary versus geriatric care. Here, we report on measures of implementation that were assessed qualitatively via focus groups and interviews with the clinics’ personnel and a survey of the clinics’ patients and care partners. We also report associated costs and efforts.

### Survey

The survey was constructed to understand patients’ and care partners’ attitudes towards shared access, experience registering for and using shared access, and perceptions of the demonstration. (see Supplemental Material 3). The survey questions were informed by the team’s prior research work regarding the patient portal, caregiving, and shared access. The post-demonstration anonymous survey was distributed electronically through eligible patient portal accounts, specifically to those patients who were: (1) age 65 and older, (2) alive at the end of the demonstration period, and (3) had one or more evaluation and management visits during the 12-month demonstration period. The survey link was also sent to care partners of those eligible patients who had shared access accounts. At organization 3, at least two visits were required for eligibility due to organizational policies regulating patient portal messaging for research. By reviewing the prospective agreement and explicitly indicating their willingness, all survey respondents consented to participate. Those responding to the survey had an opportunity to identify themselves as a patient or a care partner of the patient, regardless of whether they were responding to the survey link sent to the patient or the shared access portal account. The link led to an external survey database ensuring anonymity. Participation in the survey was not compensated. The survey was in English and contained 9 questions for care partner respondents (7 multiple-choice and 2 open-ended), 7 questions for patient respondents (5 multiple-choice and 2 open-ended), and 5 demographic questions for everyone. Only open-ended responses were analyzed in this study. Open-ended responses from the survey were extracted for each organization for each question, so that each extract could be coded separately. The survey was approved by the Johns Hopkins Bloomberg School of Public Health Institutional Review Board and the Institutional Review Boards at all 3 partner organizations (determined as exempt).

### Focus groups and interviews

All focus groups with clinic personnel were conducted during the allotted time of regularly held clinic, staff, or clinician meetings so that the meeting participant composition determined focus groups and ensured the inclusion of all types of personnel. All interviewed consented to participate and were provided with an opportunity to opt out. Anonymous participation was not possible. The meetings took place in-person, virtually, or hybrid. Some clinic personnel participants were sharing the virtual attendee accounts or were sharing their offices and office computers with each other for those meetings, which sometimes impeded attributing some remarks to a specific person. The transitions from the regular clinic meeting to focus group interviewing, hybrid or virtual participation, and account sharing precluded us from collecting participant demographics without introducing additional burden and disruptions. The focus groups were led by a qualitative health services researcher (CMD) using semi-structured interview guides mid-demonstration (approximately 6 months) and post-demonstration (approximately 12–15 months) (March 2023 to January 2024). The dates were chosen based on the feasibility of joining scheduled meetings and varied due to the different start dates of the demonstration by different clinics. The interview guides with probes were iteratively developed by the research team to align with domains of the Consolidated Framework for Implementation Research (CFIR) (see below) and elicit participant experience of implementing the demonstration (see Supplemental Materials 2 for interview guides). When important key informants (for example, physician or staff clinic champion, or health information technology specialist) were not present at the focus groups or additional input was required, the research team conducted individual interviews using modified semi-structured interview guides. The interviewer took detailed notes during both focus groups and individual interviews. All participants agreed to be interviewed and video recorded. Participants did not receive compensation. The recordings were transcribed and merged with time-corresponding commentaries left by the participants in chats. The interviews and focus groups were approved by the Beth Israel Deaconess Medical Center Committee on Clinical Investigations and the Institutional Review Boards at all 3 partner organizations and were determined to be exempt.

### Identification of costs and efforts

To estimate costs associated with the implementation of our demonstration, staff were asked to record and report on the time (in minutes) and effort (low, medium, high) spent at each clinic to deploy educational materials. We recorded expenses on printing and shipping of the materials to clinic locations. We additionally asked clinic champions to estimate their efforts (low, medium, high) associated with the clinic-wide introduction of the demonstration.

### Analysis

We used rapid assessment procedures as our method of analyzing and synthesizing input from focus groups, interviews, and the survey [24, 25]. This flexible but rigorous approach to qualitative data analysis is appropriate for studies that are conducted over a relatively short time frame with a small number of specific research questions. The qualitative team (CMD and VD) summarized three transcripts and survey extracts using a coding schema template and met to resolve discrepancies while revising the schema. This process was repeated for two more transcripts and survey extracts and was discussed until consensus was reached on the schema. The remaining transcripts and extracts were summarized by one member (VD) using the finalized coding schema and checked by another member (CMD) against their notes (See Supplemental Materials 1). The team met regularly to resolve questions and coding discrepancies. When all transcripts and extracts were summarized, a matrix was created to organize information using CFIR implementation outcomes and implementation characteristics.

### Consolidated Framework for Implementation Research

The CFIR is one of the most comprehensive and widely used frameworks for examining barriers and facilitators to implementation [26, 27]. This framework has been guiding the demonstration from the pre-demonstration collection of implementation characteristics to post-demonstration assessments of implementation outcomes. The acceptability of the demonstration and recommendations for improvements were evaluated at the 3-month point of the demonstration and informed minor modifications. We also assessed quantitatively the adoption and sustainability of the demonstration, which are reported separately [23]. Here, we focus on the remaining CFIR implementation outcomes, namely appropriateness, usefulness, and efficiency of the demonstration, including its costs. We additionally report on organizational, clinic, and patient implementation characteristics. For both implementation outcomes and characteristics, we identify facilitators and barriers of the demonstration.

## Results

### Focus groups and interviews

A total of 11 focus groups and 10 individual interviews were conducted; 5 mid-demonstration and 16 post-demonstration (Table 1). Key informants included physicians, nurses, social workers, care managers, patient-facing staff members of various roles, clinic administrators, and health information technology (IT) specialists who directly oversee the patient portal and IT help desks. The number of focus group participants in each group ranged from 5 to 25; the duration of focus groups and interviews ranged from 15 to 30 min.

### Survey respondents and comments

A total of 1,626 patients and 89 care partners across all five clinics responded to the survey (Table 2). The overall response rate was 15.1%. Of those, 308 patients left free-text comments about their experience and attitude towards granting shared access (18.9% of those who responded). To characterize those whose comments were analyzed, only 22 (7%) of those patients reflected on granting shared access during the demonstration, while 100 patients (32%) recalled granting shared access prior to the demonstration. Finally, 186 patients (60%) elaborated on why they did not sign up a care partner for shared access. Fifty-eight care partners left free-text comments about their experience with shared access registration and use (65% of care partner survey respondents). Of those, only 17 (29%) were describing their experience as someone who usually logs into the patient portal using their shared access credentials, while 35 care partners (60%) were describing their experience as someone who regularly uses patient credentials, and 6 care partners (10%) as someone whose use of the login credentials varies. Additionally, 84 patients and 21 care partners left free-text comments specifically about their experience with the demonstration, either those who recalled speaking with clinic staff about shared access or who recalled receiving written materials about shared access. The free-text responses were typically brief, most often ranging from a 3-word phrase to 15 words in 3 connected sentences.

**Table 2:** Survey respondents’ demographics by respondent type and clinic affiliation

	Organization 1	Organization 2	Organization 3
	Clinic A	Overall Clinics B-D	Clinic E
Participants messaged (n)	498	8092	3060
Response rate (%)	13.0%	15.8%	13.4%
Survey respondents (n)	75	1392	433
Patients (n)	60	1222	344
Average age (years)	77	73	75
Education:			
No college (n,%)	9 (15%)	102 (9%)	25 (7%)
Some college (n,%)	18 (30%)	404 (33%)	66 (19%)
College degree (n,%)	30 (50%)	700 (57%)	248 (72%)
No response (n,%)	3 (5%)	16 (1%)	5 (2%)
Primary language:			
English (n,%)	56 (94%)	1195 (97%)	335 (97%)
Spanish (n,%)	-	3 (1%)	1 (1%)
Other (n,%)	1 (1%)	15 (1%)	5 (1%)
No response (n,%)	3 (5%)	9 (1%)	3 (1%)
Care partners (n)	5	39	45
Spouse (n,%)	1 (20%)	11 (29%)	20 (45%)
Adult child (n,%)	3 (60%)	19 (49%)	19 (44%)
Other family (n,%)	1 (20%)	8 (21%)	5 (10%)
Other non-family (n,%)	-	1 (1%)	1 (1%)
Average age (years)	68	69	68
Primary language of care partner and patient:			
Both English (n,%)	4 (80%)	32 (86%)	34 (77%)
English for care partner only (n,%)	1 (20%)	7 (14%)	10 (22%)
No response (n,%)	-	-	1 (1%)
Unspecified survey respondent (n)	10	131	44

### Findings overview

Below, we report our findings synthesized across all data sources around barriers and facilitators of implementation outcomes: (1) appropriateness of the demonstration; (2) usefulness of the demonstration; (3) efficiency of the demonstration, including costs and efforts. We further report (4) organizational (5), clinic, and (6) patient implementation characteristics presenting those as either facilitators, barriers, or both.

## Appropriateness

### Demonstration is a part of the routine workflow

Clinicians and staff indicated several ways in which invitations to grant shared access, discussions of shared access, and offering help with granting shared access have become a part of the clinics’ routine workflows. The familiarization with shared access was done within a new patient visit, when discussing the patient portal overall and the role of care partner(s) in patients’ care, during phone call conversations with patients calling into a clinic, or by including the brochure about shared access in information sent to new patients.

#### Facilitators

Additional triggers for raising awareness of shared access were instances of care partners’ use of patient identity credentials when sending messages, or care partners communicating about patients from their personal portal accounts. Furthermore, advance care planning conversations were named as an opportunity to discuss shared access, as were case-by-case discussions with a social worker. This routinization of shared access discussions more often involved care partners if the care partner accompanied the patient to the visit or the patient was a non-English speaker or displayed signs of cognitive impairment.

*“When I am seeing patients*,* either family members or patients who are frequently contacting me through [patient portal]*,* or if I am establishing care with a really complicated patient who I know is not going to be able to manage [patient portal]*,* I will take the opportunity during the first visit to try to educate them about that [shared access].”* (physician, clinic E).

*“I find it very easy to hand out the advanced directive forms and then*,* hey*,* think about the shared access and give it[brochure] to them. And if they have questions and*,* it depends on where they are on the life trajectory… and with cognitively impaired folks*,* if the family is in the room and I know they are safe to know. That [shared access] is a really important point then. Then I also bring it [shared access] up and talk to both of them and first to the patient to get concurrence*,* and then to the potential [shared access] proxy*.” (physician, clinic B).

#### Barrier

Some clinicians noted that the topic of shared access does not come up at every visit and few patients bring this up organically:

*“I do not see patients and family members bringing it up. I think that the default is… my sense is that family members just want to keep flying under the radar and just using mom’s access if they can.”* (physician, clinic E).

### Demonstration is supported by organizational infrastructure and processes

#### Facilitators

Clinicians and staff noted being supported by organizational infrastructure, such as IT help lines handling the shared access registrations or the clinic’s capability to offer help with registration while patients and care partners are in the clinic. Organizational processes also supported the demonstration. For example, during the demonstration, shared access became included in training of new clinic staff and in the responsibilities of a dedicated staff champion. Clinicians’ knowledge about the procedures for shared access in the clinic and delegation of shared access to staff were noted as other supportive organizational processes. In another clinic, the clinician champion was viewed as an important support component for the culture change around shared access.

*“I would echo what [my doctor]* *said and that it really ends up falling on the MAs [medical assistants] and our nursing staff to actually get it over the finish line.”* (physician, clinic E).

*“Usually they [patients] are able to establish shared access with just the brochure. But we do offer in the clinic*,* if you have any issues at home at your next appointment we can assist you creating it [shared access]. Usually the appointment is very long*,* so they do not want to do it that day*,* and we provide the brochure. Usually*,* they are able to do it on their own… [but] we make that very clear… open to help.”* (medical assistant, clinic A).

### Demonstration is well received by patients and care partners

#### Facilitator

Patients and care partners noted that the process for granting and gaining shared access was easy.

*“It was set up by the staff at [clinic C]. Very easy to use.” “It was so easy that I do not remember any issues”* (patients, clinics C and D).

“*Signing up for the proxy access was easy*,* because my daughter did it for me*,* if my memory service me right.” “My two daughters signed up with no problems. The process was easy*,* and we did it together*.” (patients, clinic E).

*“I have proxy access to my wife’s account who uses another clinic location and whom I am her guardian.” “No problems singing up. My husband signed the proxy*,* so his chart would be available to me*,* so if he is not able to access it*,* I can.”* (care partners, clinics B and C).

*“Since our marriage 5 years ago*,* we each have access to each other’s account by logging in on our personal account and clicking on our spouse’s image.” “I have proxy for two others. It is simple to switch between different responsibilities.”* (care partners, clinic E).

## Usefulness

### Demonstration components that are perceived as useful

Clinicians and staff described as useful the demonstration’s brochures, poster, text about shared access generated in after-visit summaries, visit notes, and for the patient portal messaging using the smart phrase.

#### Facilitators

Clinicians and staff noted that the poster in the room helps with setting up a conversation during the visit including by referring to the poster’s slogan “People remember less than half of what their doctors say.” Others noted that the brochure is clear and concise in explaining the shared access concept, process of granting it, and its benefits. Some mentioned that the brochure’s QR code is useful in directing patients and care partners to the organization’s website resource and that they saw how the QR code was used.

*“And then we have the poster right in the room*,* which is very nice to be able to point and say*,* we will give you a brochure.”* (medical assistant, clinic A).

*“And I am often having people [with their] after visit summaries. And so usually if it has been requested*,* the code will show up there*,* sign up… and I will often give instructions with that*,* or I will point out the helpline number. And I find that [my staff colleagues] are actually really good about that as well. Really highlighting [patient portal] help number*,* or how to get signed up for [patient portal] whenever they give the summaries.”* (case manager, clinic E).

Patients and care partners also recalled their interactions with demonstration components or staff talking about shared access as positive.

*“The entire staff at [clinic D] is awesome. All I have to do is call or send a message and they answer immediately. They are always very informative about all the aspects of using [patient portal].” “she [staff member] was knowledgeable and made it easy to understand*,* offered to assist if needed.”* (patients, clinics B and D).

*“Positive experience with [clinic E] staff.” “I had a detailed question and answer session with the clinic staff.”* (patients, clinic E).

*“[I recall] instructions to create my own account rather than use my mother’s log in. Staff was knowledgeable and made sure I had my own log in.”* (care partner, clinic E).

*“Doctor and staff mention it routinely for AVS (after-visit-summary)*,* test results*,* as an option for non-urgent communication*,* etc.”* (care partner, clinic A).

### Demonstration components that are not perceived as useful

Clinicians and staff shared that they found some components of the demonstration less useful. Specifically, they acknowledged not using or discontinuing the use of staff tips sheets and clinician talking points.

#### Barriers

Clinicians and staff noted that providing information about appropriate use of shared access in the patient portal messaging either did not create traction or the staff cannot know if it led to care partners securing their own portal credentials.

*“Yeah*,* I do not know what the clinic staff would say*,* but just in my experience of following up with people… did you get this set up? I see that they have received that message*,* but then they have not set up the shared access”.* (clinician, clinic E)

Additionally, clinicians and staff shared that following the demonstration’s procedures was a part of the culture for some clinicians, but their adherence varied:

*“It [shared access] is part of my culture now… the difference*,* though*,* is whether… they [other team members] are doing it too*,* but probably at not the same level of compliance. And then the other doctors probably are not doing it the same level of compliance”.* (physician champion & clinic leader, clinic A)

Finally, several patients recalled their interactions with demonstration components or staff with negative connotations:

*“My daughter said she knew how to get shared assess because the information is in [patient portal]. The clinic may have spoken to her but did not say anything to me. I would have appreciated*,* if they had spoken directly to me.” “At a cardiology appointment*,* I was told we needed mom to re-assign consent for me to access her records. Filled it out there. Next time I took her to [clinic E]*,* I was told that it was not uploaded*,* and we needed to fill out the paperwork again. We did it twice.”* (patient and care partner, clinic E).

*“My husband did not sign up*,* because he could not even get his own [patient portal] account set up and would have been very frustrated trying to access mine*.” (patient, clinic B).

## Efficiency

### Demonstration is perceived as undemanding

Clinicians and staff reported that they did not experience increases in their personal workload due to the demonstration. They also did not see changes in the volume of messaging to them on the patient portal attributable to the demonstration.

#### Facilitators

Clinicians and staff noted that describing shared access registration did not take much of their time, generally no more than one minute to cover during rooming. They described the shared access conversation as different than the many other questions patients are asked, which helps to engage patients and care partners. Several noted that conversations about shared access are very quick because care partners often agree right away.

*“We have to go through so many steps when somebody comes and walks into the room from asking their birthday for the twentieth time. All the questions. The distress screen… They have gone through that 5 times this week. So*,* it [shared access] is like another thing to do*,* but they may not (have) heard it yet. It is like they have done all those other things before… in it [shared access] always interested. So*,* it is an extra step. But it is different than what I think they are doing. It is different.”* (patient-facing staff member, clinic A).

Health IT team leaders at all 3 systems reported that their IT teams did not experience notable changes in the volume of health IT support calls from care partners and patients or in the volume of online form requests they received. They did not report perceiving increased team workloads overall. One health IT team leader noted that because of the demonstration their organization has implemented a paperless option to register for shared access for care partners who are also patients at their organization, thus, decreasing efforts needed for shared access registration for all involved parties.

*“Because we already had [patient portal] built out to support appropriate shared access relationships*,* there was no extra work for the [patient portal] team*,* other than answering the odd question here or there. Nothing significant. Minimal impact to the Help Desk as well.”* (health IT manager overseeing support, clinic E).

#### Barrier

Clinicians noted that in the context where family dynamics are not straightforward, shared access might bring those conversations and challenges into patient portal messaging. In those cases, maneuvering through such family dynamics might take more time and effort.

*“I have one patient whom I have known for many*,* many years*,* and we had to set boundaries with her sister. She does have shared access*,* and the patient granted that to her a while ago. And we get messages from her*,* and we continually say*,* Okay*,* we will talk about this at the next visit… but the sister drove it so much that the patient shrunk away. So*,* we are a little bit more vocal on protecting that boundary.”* (physician, clinic A).

### Demonstration is low-cost

Responsible personnel reported minimal time (within an hour) and effort (low intensity) associated with the deployment of educational materials at the launch of the demonstration. Preparations for and introductions of the demonstration at all-clinic meetings or team huddles (medium intensity effort) took at most one hour of the dedicated champion’s time. The costs associated with printing and shipping bilingual educational materials for the demonstration’s launch ranged from $313 to $808 per clinic (clinics serving from 500 to 12,000 older adults annually). Additional expenses for each additional language of the educational materials ranged from $30 (one language) to $175 (four languages). Per year costs of replenishing educational materials are estimated at $100 to $260 per clinic. While commenting on the potential burden that rolling out the demonstration has placed on them, respondents noted:

*“No burden - just wanting to make sure the emphasis of the project is understood and communicated.” (clinic administrator*,* clinic B)*.

*“This roll out has not been burdensome once we established the correct workflow for assisting patients with getting shared access via [patient portal]. Compared to other pilots very easy for the staff.” (clinic administrator*,* clinic D)*.

## Organizational characterisics

### Facilitators

Clinicians and staff expressed the feeling that shared access is the right and legal thing to do, while it is wrong to log in under another’s name. They speculated that their feeling might be supported by HIPAA (The Health Insurance Portability and Accountability Act) requirements or regulations by the EHR vendors who were viewed by some as operating and setting the rules for the patient portal. Some staff indicated that they use a dot phrase with language on legal requirements to encourage patients to comply with using shared access, and one health IT specialist indicated that care partners messaging in their own charts about patients is not allowed and triggers the health IT team’s response with information about shared access.

### Privacy as both facilitator and barrier

Clinicians and staff expressed conflicting opinions as to whether shared access advances or impedes patient privacy. Not enough knowledge about how to navigate privacy of messaging with care partners and with patients at the same time, or how to navigate multiple family members using shared access or difficult family dynamics, were seen as impediments. Instances of abuse, where a care partner logs in as a patient, were seen as possibly alleviated with shared access, yet care partners might propagate and exacerbate their abuse using their shared access rights. Privacy considerations were additionally muddled by confusion with policies that regulate child and adolescent patient portals and parents’ portal access and privacy of messaging with clinicians. Additionally, some clinicians and staff who had personal experience with shared access for their loved ones on other health systems’ patient portals acknowledged being confused about how privacy is handled at those organizations versus shared access at the clinics where they work. Finally, several patients shared that they did not register their care partners due to privacy concerns (Table 3).

**Table 3:** Illustrative quotes on facilitators and barriers to shared access from patients and care partners

	Patients	Care partners
Facilitators - alignment of shared access with both patients’ and care partners’ needs	*“It gives him [care partner] the same information that I have concerning my health and his.”* (patient, clinic B) *“My husband and adult children wanted to understand health issues I have been going through.”* (patient, clinic C) *“It has been a very good experience for my husband and I to share medical information about each other and be able to communicate with each other’s medical providers.”* (patient, clinic E)	*“It has been a great tool in navigating my husband’s health care as well as communication between care providers.”* (care partner, clinic B) *“I am signed up as a [shared access] proxy for [name redacted] with dementia. It is helpful to chat with her care team privately sometimes.”* (care partner, clinic D) *“My father has Alzheimer’s and my mother does not like technology*,* so I am the main user for him. I love the easy access and the ability to review notes and tests and then explain them to my mother.”* (care partner, clinic D)
Barriers– impeding shared access use	*“My husband and I signed up for each other pretty recently. Today we decided to stop it. It was confusing and unnecessary for us to see everything on each other’s chart. Once I thought I was on my husband’s chart when indeed I had signed into mine.”* (patient, clinic C) *“My wife is the only person I would offer access*,* and I just share my login credentials with her.”* (patient, clinic D) “I was not sure I wanted my kids to see everything. I wanted that control.” (patient, clinic U).	*“Patient does not have a computer or smart phone*,* has dementia; seemed easier to just create them an account and then I can log in with their credentials. I have had to do that as their financial power of attorney for other sites*,* so it is what I am used to.”* (care partner, clinic B) *“I have my own accounts and it is easier to use her account for her [information].”* (care partner, clinic C) *“Easier to use husband’s login credentials and no additional account to keep track of*.” (care partner, clinic E)

## Clinic characterisics

### Facilitators

Clinicians and staff indicated that the demonstration was aligned with the desire to have an identified care partner on file to connect and communicate with the clinic. They noted the importance of care partner identification for safe, respectful, and clear communication. Others emphasized the ability that shared access affords care partners in communicating with the clinic independently, yet with patient permission, including about sensitive issues. Some respondents noted that proactive identification of care partners is an investment that saves time for subsequent interactions and is a component of investing in a robust structure to support age-friendly care.

### Barriers

Clinicians’ competing priorities and patient needs that must be addressed during clinic visits were named as the most notable barriers. Further, some clinicians stated that discussing shared access during a visit was not an appropriate use of their time and level of expertise.

## Patient characterisics

### Facilitators

Patients indicated that shared access supports their needs for information sharing and delegating communications with clinicians to their care partners (Table 3). Care partners similarly noted that shared access meets their needs for information and communication (Table 3).

### Barriers

Patients and care partners expressed the persistent belief that patient credentials are good enough for what they need on the patient portal. Some indicated that they were not aware of shared access or of its benefits or did not have eligible or trusted care partners. The observation that patient portal functionalities for patients and care partners do not differ also counteracted using shared access. The quotes (Table 3) convey those and additional sentiments.

## Discussion

This work reports on the perspectives of organizational and clinic personnel and patients and care partners about a first-of-its-kind demonstration to increase shared access to older adults’ patient portals. Findings suggest the demonstration was appropriate and useful, it was routinized in the clinics and well received by patients and care partners, and it was undemanding and low-cost, utilizing existing infrastructure and organizational processes. The alignment of shared access with clinic practices and philosophy, organizational policies, and the needs of patients and care partners facilitated the implementation. At the same time, numerous barriers, including clinicians’ competing priorities and patients’ and care partners’ low awareness and motivation for switching to shared access, undermined shared access uptake.

In comparison with experiences reported in existing, though limited, literature [6, 17, 18], our care partner and patient respondents indicated an overall positive experience with shared access registration, even before the demonstration. This might have been due to the selection of clinics for the demonstration that shared affinity with the Coalition for Care Partners [21], had been already promoting shared access, or had simplified their registration processes before the official launch of the demonstration [22]. However, the cumbersomeness of shared access registration is just one of the barriers behind its low uptake and use. Potentially by removing that initial barrier, we exposed other deeper barriers, such as the persistent belief that patient credentials are good enough for what care partners need on the patient portal.

All main components of the demonstration were perceived as useful, and their use together might have created synergies. At the same time, some clinician- and staff-facing components of the demonstration were utilized less often as the demonstration was routinized. This, however, does not mean that the implementation of the initiative at new clinics and organizations should forego those components. New implementation clinics might be at a disadvantage because their implementation might not be accompanied by evaluations [28]. Staff indicated that not knowing whether their efforts to promote shared access resulted in patients granting it and care partners using it was making staff participation in the demonstration less meaningful. Including frontline staff in future implementations might help to identify ways to close this loop and make staff participation more meaningful. Some staff and clinicians were informed about ongoing evaluations of the demonstration’s monthly progress. Those evaluation reports as well as the focus groups might have supported or expedited the routinization of the demonstration. Our demonstration was facilitated by either staff or clinician champions supported through the demonstration funding, and educational material costs, while low, were also covered by that funding, not by the clinics. This should be taken into account when implementing at new clinics.

Clinician and staff respondents did not perceive an additional burden of patient portal messaging attributable to the demonstration. However, the overall increase in patient portal messaging, which especially accelerated during and in the wake of the COVID- 19 pandemic, was noted and might prevent others from endorsing this initiative. Even if its undemanding nature is communicated, personnel might still be cautious of a potential increase in their workloads [29, 30]. A recent study demonstrated that visits appeared to generate new patient portal messages rather than messages replacing visits; [31] however, messaging from care partners using shared access was not studied and might have different dynamics that might add to clinicians’ concerns. A potential additional barrier that the demonstration has not encountered is a health system’s transition from one patient portal to another [32, 33]. Finally, a potential barrier that might be steering clinicians’ concerns about “misuse” of patient portals, is the lack of guidance in how to navigate communications with both patient and care partner, especially with multiple care partners and not straightforward family dynamics [34]. At the same time, the findings from this demonstration, and identified links between shared access to the patient portals of older adults and supporting age-friendly care, might facilitate the spread of this initiative [35].

Based on the mixed quantitative results [23] and barriers uncovered here, we suggest that potential solutions to promoting shared access lie in the realm of policy strategies. One strategy is to amplify benefits that using shared access can offer. For example, organizations could provide care partner-specific resources and information through shared access or add other functionalities to increase perceptions of the value of this modality of portal access. Communicating about penalties for using patients’ credentials might be another strategy. We often heard that clinicians themselves do not use shared access while executing their own care partner duties, and, thus, communicating about penalties might also be directed to clinicians. Strategies might pursue both directions– persuade and remind about penalties– at the same time. Regardless of the chosen path, serious considerations should be given to strategies that would support digital health equity for older adults, given prior research suggesting that care partners are more likely to access the portal for patients who are older, in poorer health, and less likely to speak English as a first language [10, 36].

Another potential strategy is for shared access to become an opt-out option rather than opt-in for older adults. For example, heath care organizations could synchronize naming of emergency contacts with granting them shared access by default while carefully navigating privacy and HIPAA requirements. On the other hand, raising the issues of shared access, trusted care partners, and differences between emergency contacts, healthcare proxies and shared access delegates might require engaging and understanding the patient first. The opt-out option will imply that those discussions should happen between patients, care partners, and clinicians after patients sign in to the patient portal to confirm or modify their initial choice regarding shared access. In this, shared access for older adults might learn some practices from pediatric shared access [37, 38], although legal responsibilities between parents or guardians and their children are different from legal aspects regulating health care proxies and granting and revoking shared access. The opt-out option might also require broader changes in privacy regulations. Overall, policy and implementation strategies pursuing shared access uptake among older adults should take into account considerations of the dignity and privacy of older adults and be co-designed with patients and care partners.

There were limitations to this evaluation methodology. We conducted our focus groups utilizing time that was generously allotted to us out of regularly scheduled clinics’ staff and clinician meetings. This allowed us to ensure high rates of participation and representativeness, a natural atmosphere, and minimal invasiveness of our study. However, this limited the duration of the focus groups and ability for each participant to provide detailed answers. Additionally, this might have evoked power and hierarchical dynamics among the participants and between the participants and researchers. For example, as staff were reporting to the research team on their demonstration deployment burden, they might have underestimated it. Our recruitment approach also precluded us from collecting demographic information about the focus group participants and a reliable collection of exact numbers of participants in each group and their roles. Consequently, we have not collected information about our individual interviewees, beyond their role at the clinic and in our demonstration. Furthermore, we acknowledge that our chosen recruitment strategy for the online survey, using messaging via the patient portal, as well as our response rates introduced biases in responses making them not representative of the clinics’ populations. Consequently, we have not compared demographics of those survey respondents who left open-ended responses versus those who did not since we realize the inherited challenge of representativeness in the survey. Importantly, our survey was distributed only in English, and we have not collected race and ethnicity information, acknowledging that due to our recruitment method we will not have representativeness to reliably analyze responses by these demographics. Additionally, the clinics where the demonstration took place were urban and also had relatively high patient portal activation rates. In future studies, it is important to understand perspectives on shared access from users who live in rural areas and from those who are less exposed to the patient portal overall. We also excluded from the survey patients who died during the demonstration, thus omitting perspectives of care partners who might have sought access to patient portals after the death of their loved ones and whose use of the patient portal during end-of-life care is known to be different [39].

## Conclusion

The Coalition for Care Partners has in its arsenal an appropriate, useful, undemanding, and low-cost initiative to increase shared access to older adults’ patient portals. However, additional barriers exist to the widespread implementation of this initiative to other clinics and healthcare delivery organizations. Given the strong alignment of shared access with patient- and family-centered care, future efforts might be supported by policies that recommend making shared access an opt-out choice during patient portal registration and identification of care partners. Other potential policy directions are amplifying benefits and functionalities of shared access and communicating any penalties for and potential harms of care partners unidentifiably accessing patient portals using patient credentials.

## Supplementary Information

Supplementary Material 1.

Supplementary Material 2.

Supplementary Material 3.

## Data Availability

No datasets were generated or analysed during the current study.

## References

[1] Johnson C, Richwine C, Patel V. Individuals’ access and use of patient portals and smartphone health apps, 2020. Washington, DC: Office of the National Coordinator for Health Information Technology; 2021.

[2] DHHS 2019; Pages. 2020. Accessed at DHHS at https://www.healthit.gov/playbook/pe/chapter-4/ on 2 Oct 2020.

[3] Burgdorf JG, Fabius CD, Wolff JL. Use of provider-sponsored patient portals among older adults and their family caregivers. J Am Geriatr Soc. 2023;71(4):1177-87. 10.1111/jgs.18187. 10.1111/jgs.18187 PMC10089953 36573382

[4] Antonio MG, Petrovskaya O, Lau F. The state of evidence in patient portals: umbrella review. J Med Internet Res. 2020;22(11):e23851. 33174851 10.2196/23851 PMC7688386

[5] Zulman DM, Nazi KM, Turvey CL, Wagner TH, Woods SS, An LC. Patient interest in sharing personal health record information: A Web-Based survey. Ann Intern Med. 2011;155(12):805–10. 22184687 10.7326/0003-4819-155-12-201112200-00002

[6] Latulipe C, Quandt SA, Melius KA, Bertoni A, Miller DP Jr., Smith D, et al. Insights into older adult patient concerns around the caregiver proxy portal use: qualitative interview study. J Med Internet Res. 2018;20(11):e10524. 30389654 10.2196/10524 PMC6240158

[7] Salmi L, Peereboom D, Dorr DA, Graham LR, Wolff JL, Desroches CM. Patient portals fail to collect structured information about who else is involved in a person’s care. J Med Internet Res. 2024;26:e49394. 38935963 10.2196/49394 PMC11240061

[8] NASEM. Families caring for an aging America. Washington, DC: National Academies of Sciences, Engineering, and Medicine; 2016.

[9] Riffin C, Wolff JL. Identifying, assessing, and supporting family caregivers in health and long-term care: current progress and future opportunities. In: Gaugler JE, editor. Bridging the family care gap. Elsevier; 2021. p. 341–60.

[10] Wolff JL, Dukhanin V, Burgdorf JG, DesRoches CM. Shared access to patient portals for older adults: implications for privacy and digital health equity. JMIR Aging. 2022;5(2):e34628. 10.2196/34628. 10.2196/34628 PMC9118085 35507405

[11] Wolff JL, Darer JD, Larsen KL. Family caregivers and consumer health information technology. J Gen Intern Med. 2016;31(1):117–21. 26311198 10.1007/s11606-015-3494-0 PMC4699991

[12] Casillas A, Cemballi AG, Abhat A, Lemberg M, Portz JD, Sadasivaiah S, et al. An untapped potential in primary care: Semi-Structured interviews with clinicians on how patient portals will work for caregivers in the safety net. J Med Internet Res. 2020;22(7):e18466. 32706709 10.2196/18466 PMC7400036

[13] Iott B, Raj M, Platt J, Anthony D. Family caregiver access of online medical records: findings from the health information National trends survey. J Gen Intern Med. 2021;36(10):3267-9. 10.1007/s11606-020-06350-8. 10.1007/s11606-020-06350-8 PMC7710154 33269423

[14] Wolff JL, Berger A, Clarke D, Green JA, Stametz RA, Yule C, et al. Patients, care partners, and shared access to the patient portal: online practices at an integrated health system. J Am Med Inf Assoc. 2016;23(6):1150–8. 10.1093/jamia/ocw025 PMC11960767 27026614

[15] Reed ME, Huang J, Brand R, Ballard D, Yamin C, Hsu J, et al. Communicating through a patient portal to engage family care partners. JAMA Intern Med. 2018;178(1):142–4. 29159402 10.1001/jamainternmed.2017.6325 PMC5833508

[16] Gleason KT, Peereboom D, Wec A, Wolff JL. Patient portals to support care partner engagement in adolescent and adult populations. JAMA Netw Open. 2022;5(12):e2248696. 36576738 10.1001/jamanetworkopen.2022.48696 PMC9857556

[17] Nazi KM, Turvey CL, Klein DM, Hogan TP. A decade of veteran voices: examining patient portal enhancements through the Lens of User-Centered design. J Med Internet Res. 2018;20(7):e10413. 29991468 10.2196/10413 PMC6058093

[18] Latulipe C, Mazumder SF, Wilson RKW, Talton JW, Bertoni AG, Quandt SA, Arcury TA, Miller DP Jr. Security and privacy risks associated with adult patient portal accounts in US hospitals. JAMA Intern Med. 2020;180(6):845-9. 10.1001/jamainternmed.2020.0515. 10.1001/jamainternmed.2020.0515 PMC7199170 32364562

[19] Ramirez-Zohfeld V, Seltzer A, Xiong L, Morse L, Lindquist LA. Use of electronic health records by older adults, 85 years and older, and their caregivers. J Am Geriatr Soc. 2020;68(5):1078–82. 32159860 10.1111/jgs.16393

[20] Pecina J, Duvall MJ, North F. Frequency of and factors associated with care partner proxy interaction with health care teams using patient portal accounts. Telemed J E Health. 2020;26(11):1368–72. 31971889 10.1089/tmj.2019.0208

[21] Engaging family care partners through shared access to the electronic health record. Coalition for Care Partners. 2023. https://coalitionforcarepartners.org/engaging-family-care-partners/ [accessed 2023-11-07].

[22] Dukhanin V, Wolff JL, Salmi L, Harcourt K, Wachenheim D, Byock I, et al. Co-Designing an initiative to increase shared access to older adults’ patient portals: stakeholder engagement. J Med Internet Res. 2023;25:e46146. 37991827 10.2196/46146 PMC10701652

[23] Gleason K, DesRoches C, Wu M, Peereboom D, Dukhanin V, Wolff JL, et al. Evaluation of a Multi-Site implementation study to increase proxy access to the patient portal among older adults. JAMA Netw Open. 2025;8(2):e2461803. 39998827 10.1001/jamanetworkopen.2024.61803 PMC11862967

[24] McNall M, Foster-Fishman PG. Methods of rapid evaluation, assessment, and appraisal. Am J Evaluation. 2007;28(2):151–68.

[25] Taylor B, Henshall C, Kenyon S, Litchfield I, Greenfield S. Can rapid approaches to qualitative analysis deliver timely, valid findings to clinical leaders? A mixed methods study comparing rapid and thematic analysis. BMJ Open. 2018;8(10):e019993. 30297341 10.1136/bmjopen-2017-019993 PMC6194404

[26] Rojas Smith L, Ashok M, Morss Dy S, Wines RC, Teixeira-Poit S. AHRQ methods for effective health care. Contextual frameworks for research on the implementation of complex system interventions. Rockville: Agency for Healthcare Research and Quality (US); 2014. 24783308

[27] Proctor E, Silmere H, Raghavan R, Hovmand P, Aarons G, Bunger A, et al. Outcomes for implementation research: conceptual distinctions, measurement challenges, and research agenda. Adm Policy Mental Health Mental Health Serv Res. 2011;38(2):65–76. 10.1007/s10488-010-0319-7 PMC3068522 20957426

[28] Stetler CB, Legro MW, Wallace CM, Bowman C, Guihan M, Hagedorn H, et al. The role of formative evaluation in implementation research and the QUERI experience. J Gen Intern Med. 2006;21(S2):S1–8. 16637954 10.1111/j.1525-1497.2006.00355.x PMC2557128

[29] Nøst TH, Faxvaag A, Steinsbekk A. Participants’ views and experiences from setting up a shared patient portal for primary and specialist health services- a qualitative study. BMC Health Serv Res. 2021;21(1):171. 33627122 10.1186/s12913-021-06188-8 PMC7903028

[30] Reinhardt I, Holsten R, Zielasek J, Kuhlmann L, Gouzoulis-Mayfrank E. Implementation of an electronic patient portal in routine mental health care of hospitals in Germany– evaluation of attitudes of healthcare providers. BMC Health Serv Res. 2024;24(1):1213. 39390434 10.1186/s12913-024-11686-6 PMC11465699

[31] Martinez KA, Schulte R, Rothberg MB, Tang MC, Pfoh ER. Patient portal message volume and time spent on the EHR: an observational study of primary care clinicians. J Gen Intern Med. 2024;39(4):566–72. 38129617 10.1007/s11606-023-08577-7 PMC10973312

[32] Fix GM, Haltom TM, Cogan AM, Shimada SL, Davila JA. Understanding patients’ preferences and experiences during an electronic health record transition. J Gen Intern Med. 2023. Epub ahead of print. 10.1007/s11606-023-08338-6. 10.1007/s11606-023-08338-6 37580637

[33] Ball SL, Kim B, Cutrona SL, Molloy-Paolillo BK, Ahlness E, Moldestad M, et al. Clinician and staff experiences with frustrated patients during an electronic health record transition: a qualitative case study. BMC Health Serv Res. 2024;24(1):535. 38671473 10.1186/s12913-024-10974-5 PMC11046755

[34] Lee JL, Williams CE, Baird S, Matthias MS, Weiner M. Too many Don’ts and not enough Do’s? A survey of hospitals about their portal instructions for patients. J Gen Intern Med. 2020;35(4):1029–34. 31720967 10.1007/s11606-019-05528-z PMC7174450

[35] Fulmer T, Mate KS, Berman A. The Age-Friendly health system imperative. J Am Geriatr Soc. 2018;66(1):22–4. 28876455 10.1111/jgs.15076

[36] Grossman LV, Masterson Creber RM, Benda NC, Wright D, Vawdrey DK, Ancker JS. Interventions to increase patient portal use in vulnerable populations: a systematic review. J Am Med Inf Assoc. 2019;26(8–9):855–70. 10.1093/jamia/ocz023 PMC6696508 30958532

[37] Thompson LA, Martinko T, Budd P, Mercado R, Schentrup AM. Meaningful use of a confidential adolescent patient portal. J Adolesc Health. 2016;58(2):134–40. 26802988 10.1016/j.jadohealth.2015.10.015

[38] Patel PD, Cobb J, Wright D, Turer RW, Jordan T, Humphrey A, et al. Rapid development of telehealth capabilities within pediatric patient portal infrastructure for COVID-19 care: barriers, solutions, results. J Am Med Inf Assoc. 2020;27(7):1116–20. 10.1093/jamia/ocaa065 PMC7188108 32302395

[39] Portz JD, Powers JD, Baldwin M, Bekelman DB, Casillas A, Kutner JS, Bayliss E. Patient portal use near the End-of-Life. J Gen Intern Med. 2021. Epub ahead of print. 10.1007/s11606-020-06333-9. 10.1007/s11606-020-06333-9 33506403

