# Supplementary material for: Users’ perspectives on a demonstration to increase shared access to older adults’ patient portals
Source: BMC Health Serv Res. 2025 Apr 23;25:586. doi: 10.1186/s12913-025-12755-0 (PMC12016354; doi:10.1186/s12913-025-12755-0)
Supplement: Supplementary file 1 — Supplementary Material 1. [file 12913_2025_12755_MOESM1_ESM.pdf]

Appropriateness	Usefulness	Impact & Sustainability	Facilitators & Barriers	Other
**Why does the intervention fit well?**	**What is useful?**	**What happened as a result of the intervention?**	**What helps with promoting and using Shared Access?**	Preference for paper forms to register U12CM/SW
	**Summary: Multimodal usefulness**			
[Infrastructure] There is an IT help line handling the registration U12CM/SW US12 US6	Poster is useful, for example, for setting up a conversation U12CM/SW U12CNC U12MD US12 US6 UC6 RFG12 RPA	No change in the volume of messaging U12CM/SW U12CNC U12MD RPA PSh12 PGa12	[We need it] Need for an identified care partner on file to connect and communicate with the clinic U12CM/SW RFG12	Preference for digital registration U12CM/SW
[Workflow] Part of routine care U12CM/SW US6 UC6 RFG12	Brochure is useful in explaining patients shared access and the process, it is clear and concise RPA US6	No increase in personal workload U12CM/SW	[We need it] It is important for safe, respectful, or non-confusing communication to be identified as a care partner U12CM/SW UC12 RPA RMD PGa6	Do not know about where the poster QR leads you to U12CM/SW
	Brochure is available just to hand out for patients to take home US6 PSh6 PGli6 PSh12 PGli12		[We need it] need it to be an age friendly health system, part of our investment in robust structure PCliCh	Overall increase in patient portal interactions, unrelated to shared access US6 UC6
	Brochure has a convenient QR code UC6 PGli6			One of the clinics stopped accepting new patients during the intervention US6
[Workflow] Part of a new patient visit U12CM/SW UC12	Shared access info can be generated in after-visit summary U12CM/SW UC12 UC6	Seeing more messages from care partners using shared access credentials U12CM/SW U12MD UC12 UC6 RPA PGli6	[We need it] Shared access is a way for a care partner communicate with the clinic independently, with patient permission, sometimes about sensitive issues RFG12 RPA	Use of portal messaging mundanely, not for medical issues U12CM/SW UC6
[Workflow] Part of phone conversations US6				
[Workflow] Part of social worker procedures, informally and case-by-case U12CM/SW US6 RSW	Having a dot phrase as a tool that provides a script U12CNC US12 US6	Seeing more patients that have shared access set up RMD UC6	[We need it] Pointing out to patients that it [shared access] is important U12CNC UC12	Use of portal messaging for urgent issues instead of having appointments U12CM/SW UC12
[Infrastructure] Part of the training of clinical staff which was not before U12CNC US6 UC6	Does not take much time U12MD UC12 US12 PGa6 PGa12 PGli12	Can sometimes take a lot of time and bring in family dynamics UC12 RMD	[We need it] It reflects the needs of our patient population U12CNC PSh12	Education of patients and care partners on patient portal messaging U12CNC
[Workflow] Part of a new patient packet that is sent out U12CNC US6	**What is not useful?**	[Does not take much time] It does not take more than one minute to cover shared access during rooming RPA PGa12	[Alignment] Speculating that patient portal is governed by an EHR vendor so granting shared access is separate from our health system's forms U12CNC	Poster is used a teaching tool for trainees how to communicate with patients UC6
[Workflow] Part of advance care planning conversations UC6 PGa6				

[Infrastructure] Shared access procedures are delegated to staff away from clinicians UC12 UC6 US6 PSh6 PGa12	I do not use tip sheets U12CM/SW US6	[Does not take much time] The conversation about shared access is very quick as care partners agree right away PSh12	[Alignment] Dot phrase with language on legal requirement to comply with using shared access not patient credentials U12CNC US12 US6 UC6	Patient portal allowing to share hyperlinks and other attachments versus over the phone UC6
[Part of routine care, subtheme] It sometimes come up at the visit, for some patients, but most do not bring it up organically U12MD UC12	Not much traction after using dot phrase UC12	[Does not take much time] It is not disruptive, another quick thing to do. That thing is "different" from regular clinic rooming aspects RFG12 RPA	[Alignment] Citing the health system's requirement to use shared access, due to HIPAA U12MD PGa6<br>[Alignment] Care partners messaging in their own charts about patients is obviously not allowed and triggers the IT response, dot phrase PIT	Asking to bring this intervention to a sister clinic as internal medicine has many geriatric patients US6
[Part of routine care, subtheme] It happens naturally for almost all patients as patient portal and if they have a care partner are discussed at visits RPA	[variability] Some clinicians and staff do not use educational materials UC12 US12 UC6 RFG12	We have implemented an option to invite a proxy using patient portal [no paper], if a care partner is also in the system RIT	[Mix Privacy] Considering shared access versus logging in as a patient to be a patient privacy issue, so patient can message privately RPA	
[Infrastructure] Clinicians know what the process in the clinic is U12MD UC12 RFG12 PGa12	I do not use talking points, use my own language PSh12	No change in IT health support calls volume or online forms volume requests, no extra work RIT UIT PIT	[Alignment] Feeling that shared access is the right/correct and legal thing to do, while it is wrong logging in under other's name U12MD RPA	
[Workflow] Comes up at patient portal conversations when care partners use patient login or send messages about patients from their personal portal account UC12 RIT PSh12 PGa12	**What would be good for future use and improvement?**		[We need it] Our clinic might be the first place where a care partner is identified and given shared access that helps with this patient's interactions with other doctors in our health system RFG12	
[Infrastructure] There is a dedicated staff champion RFG12<br>[Infrastructure] The role of clinical champion is important for culture change PCliCh	Have a snapshot of patient portal menu to explain what it is U12CM/SW	**Do you plan to sustain the intervention?**	Explaining patients that shared access helps to feel less overwhelmed with care RMD	
[variability] Part of the culture for some clinicians but adherence varies RFG12 RMD	Shared access would allow care partners to stay in contact with the clinic after the patient death for 6 months while the patient account is closed RFG12	We want to continue using the educational materials U12CNC U12MD RFG12 PSh12	[We need it] It is an investment that saves time for the clinic downstream RSW<br>Patient changing their password and care partner cannot longer log in as patient US6	

[Infrastructure] Clinic has a capability to offer help with registering while in the clinic RPA UC6	Including shared access in the totality of patient centered advance care planning, including using champions and fellows models PCliCh		There is an education opportunity moment when patients reach out to activate back their access deactivated because of care partners were logging in with patient credentials RIT	
[Part of routine care, subtheme] If a patient comes in with a family member, especially more than once or non-English speaker ow with cognitive issues, it seems appropriate to talk about shared access with them PSh12 PSh6 PGa12	Much of clinical champion work can be delegated to clinical team members and administrative leadership, it is a team sport PCliCh		As a provider, referring to personal experience being a care partner who uses shared access for their loved ones PSh12 PSh6 UC6	
[Part of routine care, subtheme] During primary care visit or when a provider prepares for that visit, they might come up with realization that they should be taking with a family member PSh12			[preference to this from patients' survey directly] Shared access is something that care partners and patients are really looking for PSh12	
			**What impedes promoting and using Shared Access?**	
			Competing priorities during the visit, not enough time U12CNC UC12 US12 US6 PGli6	
			Difficult to explain shared access to patients with diminished capacity and/or cognitive impairment UC6	
			Some clinicians do not see talking about shared access as the best use of their time and expertise U12MD UC12	
			[Mix Privacy] Considerations of abuse, though a care partner not being in control of patient account, as they use shared access, might alleviate U12MD RPA RSW PGa6 PGa12	
			Staff not knowing if their discussions with patients resulted	

			in patients signing up for shared access or not US6	
			Patient credentials for log in is good enough US6 PGa6 PSh6 PGa12 (+ surveys R P U)<br>Especially if an issue is important, providers will respond any way, undermining the need to have own credentials US6<br>Care partners receiving the same information whether logging in as a patient or as through shared access UC6	
			[Mix Privacy] Providers not knowing how to navigate privacy of messaging with care partners and with patients at the same time UC6	
			[Mix Privacy] Providers not knowing how to navigate multiple family members using shared access or difficult family dynamics PSh6 PGa12	

21 focus group or individual interview

U12CM/SW<br>U12CNC<br>U12MD<br>UC12<br>US12<br>UIT	RFG12<br>RPA<br>RMD<br>RSW<br>RIT	PSh12<br>PGa12<br>PGli12<br>PIT<br>PCliCh
UC6<br>US6		PGa6<br>PSh6<br>PGli6
