# Supplementary material for: Users’ perspectives on a demonstration to increase shared access to older adults’ patient portals
Source: BMC Health Serv Res. 2025 Apr 23;25:586. doi: 10.1186/s12913-025-12755-0 (PMC12016354; doi:10.1186/s12913-025-12755-0)
Supplement: Supplementary file 2 — Supplementary Material 2. [file 12913_2025_12755_MOESM2_ESM.pdf]

Supplemental Materials.

I. Interview guide for Post-demonstration focus groups with **Clinicians and Staff**

This is a short reminder that since July 2022 XXX clinic has been participating in the shared access initiative. The initiative is focused on increasing the number of care partners registered for shared access to the patient portal for patients who are seen in this clinic. This shared access is sometimes also called proxy access. The initiative includes patient- and family-facing brochures and posters in your clinic, tip sheets available to staff, talking points and dot/smart phrases available to clinicians about shared or proxy access to the patient portal for care partners. The goal is to increase awareness and explain benefits to care partners of older adults of having their own shared access account to log into the patent portal under their own credentials. This initiative is also supported by a web page at your health system web site and the evaluation of MyChart registration and use using your health system's EHR data. We talked to you about this initiative a couple of times before and would like to look back on any changes that have transpired since.

- Do you have a process as part of your regular workflow introducing and discussing shared access during patient visits? Have you used the same process since the beginning of the initiative, July 2022, or did you change it? For example, when to give a brochure, [for clinicians] when to raise the issue, or [for staff] when to offer help with the shared access registration.
- [clinician] Do you use talking points, paraphrase or describe shared access in your own words?
- [staff] Do you follow tip sheets or web materials when helping a patient to grant shared access?
- How could your workflow and materials about shared access be improved to meet your needs?
- Did you notice spending more time with patients or patients who have accompanying family members during their visit with you because you talk about shared access with them? If you are spending more time on visits, can you estimate the amount of additional time spent per visit?
- Did you notice spending more time responding to MyChart messages from care partners who use shared access? Did you notice spending less time on MyChart interactions with care partners who used to log in and interact with you as they are patients themselves? Can you quantify if there is an overall change in your time interacting with care partners on MyChart?

- Did you notice any disruptive changes in your workflow due to patients or care partners bringing up shared access more often or other shared-access-related disruptions?
- Do you envision continuing your current process around shared access at Gateway? Do you expect any changes as the formal demonstration period ends? For example, will you be requesting your administrator to print new brochures as the current supply runs out?
- If you are asked to explain why the clinic needs to continue the shared access initiative and to justify printing costs for replenishing education materials, what would you say?

II. Interview guide for Mid-demonstration focus groups with **Clinicians**

How often are you talking with patients and families about shared access to the patient portal?

Have you used any of the educational materials in conversations with patients and families about shared access to the portal?

How useful are the education materials?

Which of the materials was the most effective? Or anything is not useful? What can make it more useful?

Have you noticed any changes in how you talk about shared access with patients and families?

Have you noticed any change in the awareness of patients about shared/proxy access to the electronic health record?

Did you notice any change in the activity of patients and their care partners on MyChart with you?

How could the materials be improved?

III. Interview guide for Mid-demonstration focus groups with **Staff**

How often are you talking with patients and families about shared access to the patient portal?

Have you used any of the educational materials in conversations with patients and families about shared access to the portal?

How useful are the education materials?

Which of the materials was the most effective?

Did you notice the change in the awareness of patients about shared/proxy access to the electronic health record?

What have you heard from patients about the registration? Anything changed at all?

[Are you involved in answering MyChart messages?] [If yes] Did you notice any change in the activity of patients and their care partners on MyChart with you? More frequent use? More care partners using their own MyChart identity credentials? More care partners messaging?

How the process can be improved? Are the educational materials aligned with your process? Did you make any changes to the process that you can make?

How could the materials be improved?

IV. Interview guide for an individual interview with a **Clinical Champion**

In addition to focus group interviews with your clinic providers and staff, we would like to ask a few questions about your role as a clinical champion for the shared access initiative. Confidentiality of your responses is guaranteed.

1. Please describe your role as a clinical champion for the shared access initiative. What activities did you undertake and what were the time and effort magnitude of those commitments?
2. How likely do you think that another clinic in your health system would be able to implement the shared access initiative (with no evaluations) without a dedicated clinical champion? Could this initiative be implemented with minimal support from a clinical champion?
3. How important is the role of a clinical champion in sustaining the shared access initiative as a part of the routine care at your clinic after the demonstration period ends? Can it be sustained without associated support for that role?
4. How likely do you think that your clinic or health care system will provide support to continue the initiative after the demonstration period ends?
5. Were there other internal personnel support or resources that were critical for you in your clinical champion role?
6. In your opinion, is there an alternative designation but among clinic staff who can “own” the process of shared access initiative implementation, including planning, execution, and engagement of others?

V. Interview guide for an individual interview with a **Health Information Technology Champion**

In addition to focus group interviews with providers and staff of the clinics participating in the shared access initiative at your health system, we would like to ask a few questions about health IT support for the shared access initiative. Confidentiality of your responses is guaranteed.

However, answering these questions might require input from other heath IT personnel, such as those at helpdesk, call center, or the HIM department.

1. In your health system, since XXX 2022 XYZ clinic(s) participated in the shared access initiative. Have you noticed the change in how many requests related to shared access registration and use you had to handle? Requests from patients, their families, staff at the clinics? If there is any change, can you quantify in terms of additional personnel efforts and time?
2. Were there any other impacts of the shared access initiative at XYZ clinic(s) on health IT support at your health system (excluding evaluation-related tasks)?
3. Did you notice any differences among health IT support team (formal and informal) towards family members using patient's credentials to log into the patient portal versus using own credentials granted via shared access?

VI. Interview guide for an individual interview with a **Clinic Coordinator or Administrator**

In addition to focus group interviews with your clinic providers and staff, we would like to ask a few questions that are pertinent to your specific responsibilities at the clinic. These questions focus on staff workflow and burden and are listed below. Confidentiality of your responses is guaranteed.

1. Have you heard about any staff complaints regarding the shared access initiative, for example additional tasks or more time spent with patients on shared access registration and troubleshooting? Any other relevant complaints?
2. How has the implementation of the shared access initiative at your clinic since XXX 2022 impacted your staff and clinic administration aspects? If there is any change, can you quantify in terms of additional staff efforts and time?
3. Is the shared access initiative a part of your onboarding of new staff? Do you plan to continue to train new staff about the shared access initiative?
4. Are you aware of your clinic's plans for printing out new brochures about shared access in the future as the current supply runs out? Do you think your clinic will be able to find funds for that?
