# Supplementary material for: Users’ perspectives on a demonstration to increase shared access to older adults’ patient portals
Source: BMC Health Serv Res. 2025 Apr 23;25:586. doi: 10.1186/s12913-025-12755-0 (PMC12016354; doi:10.1186/s12913-025-12755-0)
Supplement: Supplementary file 3 — Supplementary Material 3. [file 12913_2025_12755_MOESM3_ESM.pdf]

PATIENT REDCAP SURVEY

[Text in MyChart Recruitment message]

XXX clinic is committed to helping patients and families participate actively in their care. As part of this commitment, we want to understand awareness about MyChart. You are being contacted to complete a survey to help us understand your experiences and perspectives because you are registered for the MyChart account of a patient receiving care at XXX clinic, either as the patient or as a care partner of the patient.

Answering the survey will take about 15 minutes. Your answers will be kept confidential. Your decision to take part in the survey will not affect care at XXX clinic.

If you have questions or concerns, please feel free to contact us at (CONTACT INFO). On behalf of XXX clinic, thank you!!

If you would like to respond to the survey please click here.

[Prospective agreement]

The purpose of this study is to understand awareness about MyChart. We want to understand your experiences as a MyChart user for a patient account, either as the patient or as the care partner of the patient.

Completion of the survey is voluntary. By clicking to start the survey, you are consenting to the study. You are not obligated to participate and you can stop participating at any time.

Your decision to participate in the study will not affect the care you receive at the clinic. Your answers will be kept confidential. You will not be compensated.

If you would like to start the survey, please click here.

Survey Start:

INSTRUCTION TO RESPONDENT: YOU ARE RECEIVING THIS SURVEY BECAUSE YOUR EMAIL ADDRESS IS REGISTERED TO THE MyChart account of [insert patient name]

1) **Are you**
   - The patient under the patient registered to this MyChart account?
   - Someone other than the patient.

     1a) [If someone other than the patient] **What is your relationship to the patient?**
     - Spouse
     - Adult Child
     - Other Family
     - Close Friend / Other [free text]

2) **How many times did you access your [the patient's] online medical record (MyChart) in the last 12 months?**
   - Never
   - 1-2 times
   - 3-5 times
   - 6-9 times
   - 10+ times

3-6 are only to be answered IF the answer to 1 is "someone other than the patient"

3) **How do you usually log in to the patient's online medical record (MyChart)?**
   - I usually log in as the patient, with the patient's login/password
   - I usually log in as myself
   - It varies

4) **How long have you had access your [the patient's] MyChart account?**
   - Less than one last year
   - Longer than 1 year, but less than 5 years
   - 5 or more years

5) **How did you learn about MyChart?**
   - I heard about it from the clinicians and staff at XXX clinic
   - I heard about it from clinicians and staff at a different clinic
   - Other

6) **The XXX Health System MyChart patient portal offers patients the option of giving another person, such as a spouse, adult child, or friend, access to their medical record through MyChart. This is called proxy access.**

   If you signed up for proxy access, please tell us about your experience getting signed up and using MyChart. If you did not sign up, why not? [Free text]

7-8 are only to be answered IF the answer to 1 is "I am a patient…"

7) **The XXX Health System MyChart patient portal offers patients the option of giving another person, such as a spouse, adult child, or friend, access to their medical record through MyChart. This is called proxy access. Before today, were you aware that XXX System offers proxy access?**
   - yes
   - no

7a) [If yes] **Have you ever allowed a family member or friend to sign up for proxy access to your portal account?**

- yes
- no

7b) [if yes] **What is their relationship to you?**

- Spouse
- Adult Child
- Other Family
- Close Friend / Other

7c) [if yes] **When did you grant your [relationship] access to your MyChart account?**

- Less than one last year
- Longer than 1 year, but less than 5 years
- 5 or more years

7d) Where did you learn about MyChart proxy?

- I heard about it from the clinicians and staff at XXX clinic
- I heard about it from clinicians and staff at a different clinic
- Other [Free text]

8) **If you had a care partner sign up for proxy access, please tell us about your experience with getting the care partner signed up and using MyChart. If your care partner did not sign up, why not?** [Free text]

Everyone answers 9-15

9) **In the last year at XX clinic, do you remember receiving written materials about MyChart proxy access?**
   - Yes
   - No

10) **In the last year at XX clinic, do you remember speaking to the clinicians or staff about MyChart proxy access?**
    - Yes
    - No

11) **In the last year at X clinic, may you describe any experience speaking with clinic staff about MyChart proxy access or receiving any written materials about it?** [Free text]

Demographics

12) **What is your age in years?** ___

13) In general, how would you rate your overall health?

- Excellent
- Very good
- Good
- Fair
- Poor

14) What is the highest grade or level of school that you have completed?

- Did not graduate from high school
- High school graduate or GED
- Some college credit, but no degree
- Associate's degree
- 4-year college degree
- Master's, professional or doctorate degree

15) How confident are you about your ability to take good care of your health?

- Completely confident (1)
- Very confident (2)
- Somewhat confident (3)
- A little confident (4)
- Not confident at all (5)

16 is only to be answered IF the answer to 1 is "someone other than the patient"

16) What are the primary languages spoken by you and the patient?

- My primary language is English, the patient's primary language is other than English
- My primary language is English, the patient's primary language is also English
- My primary language is not English, the patient's primary language is English
- My primary language is not English, the patient's primary language is also not English

17 is only to be answered IF the answer to 1 is "I am a patient…"

17) What is the primary languages spoken by you?

- English
- Spanish
- Other than English or Spanish
